# Supplementary material for: Lack of cortistatin drives neuroimmune and vascular dysfunction in brain ischemia
Source: J Biomed Sci. 2026 Jun 20;33:65. doi: 10.1186/s12929-026-01271-w (PMC13282875; doi:10.1186/s12929-026-01271-w)
Supplement: Supplementary file 1 — Supplementary Material 1 [file 12929_2026_1271_MOESM1_ESM.pdf]

ADDITIONAL FILE 1

Lack of cortistatin drives neuroimmune and vascular dysfunction in brain ischemia

Julia Castillo-González[1,2,3*], Pablo Vargas-Rodríguez[1], Ignacio Serrano-Martínez[1], Alejandro Cuenca-Martagón[1], Irene Forte-Lago[1], Melanie Price[2,3], Lara Buscemi[2,3], Lorenz Hirt[2,3], José Luis Ruiz[1,4,5], Elena González-Rey[1*].

[1]Institute of Parasitology and Biomedicine (IPBLN-CSIC), Granada, Spain;
[2]University of Lausanne, Lausanne, Switzerland;
[3]Lausanne University Hospital, Lausanne, Switzerland;
[4]Functional Genomics Center Zurich, ETH Zurich and University of Zurich, Zurich, Switzerland
[5]Swiss Institute of Bioinformatics, Amphipôle, Quartier UNIL-Sorge, Lausanne, Switzerland

***Correspondence**: elena.g@csic.es; Julia.CastilloGonzalez@unil.ch; Tel.: +34-958-181670

Contents

Table S1 (this file). Sequence of primers used for real-time PCR quantifications.

Table S2 (Excel file). Differentially expressed genes (DEGs) in the healthy contralateral (CL) hemisphere of *Cort*$^{-/-}$ compared to *Cort*$^{+/+}$. DEGs were ordered by FDR values. Log_2FC (fold-change expression of DEGs between *Cort*$^{-/-}$ and *Cort*$^{+/+}$ expressed as log2 values) is included. Red: upregulated DEGs in the CL hemisphere from *Cort*$^{-/-}$*;* blue: downregulated DEGs in the CL hemisphere from *Cort*$^{-/-}$*.* RPKM average from different biological replicates of the CL hemisphere from *Cort*$^{-/-}$ and *Cort*$^{+/+}$ is shown. FDR: False Discovery Rate. RPKM: reads per kilobase million.

Table S3 (Excel file). Differentially expressed genes (DEGs) in the ischemic (MCAO) hemisphere of *Cort*$^{-/-}$ compared to *Cort*$^{+/+}$*.* DEGs were ordered by FDR values. Log_2FC (fold-change expression of DEGs between *Cort*$^{-/-}$ and *Cort*$^{+/+}$ expressed as log2 values) is included. Red: upregulated DEGs in MCAO from *Cort*$^{-/-}$*;* blue: downregulated DEGs in MCAO from *Cort*$^{-/-}$. RPKM average from different biological replicates of the ischemic hemisphere from *Cort*$^{-/-}$ and *Cort*$^{+/+}$ is shown. FDR: False Discovery Rate. RPKM: reads per kilobase million.

Table S4 (Excel file). Functional enrichment analysis for ischemic *Cort*$^{-/-}$ compared to *Cort*$^{+/+}$*.* GSEA (Gene Set Enrichment Analysis) was performed using gene sets obtained from ischemic hemispheres isolated from *Cort*$^{-/-}$ and *Cort*$^{+/+}$ mice. This analysis was run for GO (sheet 1), KEGG (sheet 2), Reactome (sheet 3), and Hallmarks databases from MsigDB (sheet 4).

Table S5 (Excel file). Differentially expressed genes (DEGs) identified between healthy (CL) and ischemic (MCAO) *Cort*$^{+/+}$ hemispheres. DEGs were ordered by FDR values. Log_2FC (fold-change expression of DEGs between MCAO and CL for *Cort*$^{+/+}$ expressed as log2 values) is included. Red: upregulated DEGs in *Cort*$^{+/+}$ MCAO hemisphere compared to CL; blue: downregulated DEGs in *Cort*$^{+/+}$ MCAO hemisphere compared to CL. RPKM average from different biological replicates of *Cort*$^{+/+}$. The CL hemisphere and the *Cort*$^{+/+}$ MCAO hemisphere are shown. FDR: False Discovery Rate. RPKM: reads per kilobase million.

Table S6 (Excel file). Differentially expressed genes (DEGs) identified between healthy (CL) and ischemic (MCAO) *Cort*$^{-/-}$ hemispheres. DEGs were ordered by FDR values. Log_2FC (fold-change expression of DEGs between MCAO and CL for *Cort*$^{-/-}$ expressed as log2 values) is represented. Red: upregulated DEGs in *Cort*$^{-/-}$ MCAO hemisphere compared to CL; blue: downregulated DEGs in *Cort*$^{-/-}$ MCAO hemisphere compared to CL. RPKM average from different biological replicates of the *Cort*$^{-/-}$ CL hemisphere and the *Cort*$^{-/-}$ MCAO hemisphere is shown. FDR: False Discovery Rate. RPKM: reads per kilobase million.

Table S7 (Excel file). Gene Ontology (GO) Biological Process terms identified between healthy (CL) and ischemic (MCAO) hemispheres in *Cort*$^{-/-}$ and *Cort*$^{+/+}$*.* GO ID and definition for terms in Biological Processes are grouped into manually annotated clusters as shown in Fig. 2c. Common GO terms between genotypes (sheet 1), GO terms *Cort*$^{-/-}$ specific (sheet 2), and GO terms Cort$^{+/+}$ specific (sheet 3) are represented. Frequency: number of DEGs (FDR < 0.05) in each GO term relative to the total number of genes that are annotated in each GO term.

Table S8 (Excel file). Gene Ontology (GO) Cellular Components terms identified between healthy (CL) and ischemic (MCAO) hemispheres in *Cort*$^{-/-}$ and *Cort*$^{+/+}$*.* GO ID and definition for terms in Cellular Components are grouped into manually annotated

clusters as shown in Additional File 2: Fig. S2. Common GO terms between genotypes (sheet 1), GO terms *Cort*$^{-/-}$ specific (sheet 2), and GO terms *Cort*$^{+/+}$ specific (sheet 3) are represented. Frequency: number of DEGs (FDR < 0.05) in each GO term relative to the total number of genes that are annotated in each GO term.

Table S9 (Excel file). Gene Ontology (GO) Molecular Functions terms identified between healthy (CL) and ischemic (MCAO) hemispheres in *Cort*$^{-/-}$ and *Cort*$^{+/+}$. GO ID and definition for terms in Molecular Functions were grouped into manually annotated clusters as shown in Additional File 2: Fig. S3. Common GO terms between genotypes (sheet 1), GO terms *Cort*$^{-/-}$ specific (sheet 2) and GO terms *Cort*$^{+/+}$ specific (sheet 3) are represented Frequency: number of DEGs (FDR < 0.05) in each GO term relative to the total number of genes that are annotated in each GO term.

Table S10 (Excel file). Differentially expressed genes (DEGs) identified between healthy (CL) and ischemic (MCAO) hemispheres and shared by *Cort*$^{-/-}$ and *Cort*$^{+/+}$. Common DEGs were represented with the Log_2FC (fold-change expression of DEGs between MCAO and CL, expressed as log2 values) in *Cort*$^{+/+}$ and *Cort*$^{-/-}$.

Table S11 (this file). Differentially expressed genes (DEGs) identified between healthy (CL) and ischemic (MCAO) hemispheres common in both genotypes with stronger upregulation in *Cort*$^{-/-}$ (depicted in Fig. 2d).

Table S12 (this file). Differentially expressed genes (DEGs) identified between healthy (CL) and ischemic (MCAO) hemispheres common in both genotypes with stronger upregulation in *Cort*$^{+/+}$ (depicted in Fig. 2d).

Table S13 (this file). Downregulated differentially expressed genes (DEGs) identified between healthy (CL) and ischemic (MCAO) hemispheres common in both genotypes (depicted in Fig. 2d).

Table S14. Cell-specific gene sets (Excel file). Marker genes for major cell types in the mouse brain as described in Mancarci *et al*., 2017 (sheet 1) and Zheng *et al.,* 2022 (sheet 2). These reference gene markers were used for MGP estimations and cell-type-specific enrichment analyses (GSEA) for each experimental group in the present study.

Table S15 (this file). Quantification of neurological lesion. For each group, data indicate the absolute number and percentage of total animals with large lesion (over 30 mm^3) and significant neuronal loss (MAP2 negative area over 5 mm^2). *Cort*$^{+/+}$: *Cort*$^{+/+}$ MCAO saline-treated mice, *Cort*$^{-/-}$ + CST: *Cort*$^{+/+}$ MCAO cortistatin-treated mice, *Cort*$^{-/-}$: *Cort*$^{-/-}$ MCAO saline-treated mice, and *Cort*$^{-/-}$ + CST: *Cort*$^{-/-}$ MCAO cortistatin-treated mice.

Table S16 (this file). Quantification of neurofunctional deficits. For each group, data indicate the absolute number and percentage of total animals with no reaches and more than 5 falls from the wire-hanging test (WH), as well as animals categorized based on the time taken to descend from the pole test. *Cort*$^{+/+}$: *Cort*$^{+/+}$ MCAO saline-treated mice, *Cort*$^{-/-}$ + CST: *Cort*$^{+/+}$ MCAO cortistatin-treated mice, *Cort*$^{-/-}$: *Cort*$^{-/-}$ MCAO saline-treated mice, and *Cort*$^{-/-}$ + CST: *Cort*$^{-/-}$ MCAO cortistatin-treated mice.

- Tables S1, S11, S12, S13, S15 and S16 are included in this file and are also available as an Excel file.
- Tables S2, S3, S4, S5, S6, S7, S8, S9, S10, and S14 are too complex to be presented here and can only be available in the accompanying Excel file.

Gene	Primer sequence (5’-3’)
Adora2 (NM_001428350.1)	Fw: CACGCAGAGTTCCATCTTCAGC<br>Rv: CCCAGCAAATCGCAATGATGCC
Arg1 (NM_007482.3)	Fw: ATG GAA GAG ACC TTC AGC TAC<br>Rv: GCT GTC TTC CCA AGA GTT GGG
Bdnf (NM_001048141.1)	Fw: CCC TCC CCC TTT TAA CTG AA<br>Rv: GCC TTC ATG CAA CCG AAG TA
Cort (NM_007745.5)	Fw: GCC TTC TGA CTT TCC TTG CC<br>Rv: GAA AGC TCC CCG CTG ATT GA
Cd68 (NM_001291058.1)	Fw: CCA TCC TTC ACG ATG ACA CCT<br>Rv: GGC AGG GTT ATG AGT GAC AGT T
Cd163 (NM_001170395.1)	Fw: TGA CGA CAA CTT CAG CAA AGA<br>Rv: CCA GAA CCA GCT CCC AAT TTA
Cd5l (NM_009690.2)	Fw: GAGGACACATGGATGGAATGT<br>Rv: ACCCTTGTGTAGCACCTCCA
Cldn1 (NM_016674.4)	Fw: GAC TGT GGA TGT CCT GCG TT<br>Rv: TCA TGC CAA TGG TGG ACA CA
Cldn5 (NM_013805.4)	Fw: TAA GGC ACG GGT AGC ACT CA<br>Rv: GCC CAG CTC GTA CTT CTG TG
Col1a2 (NM_007743.3)	Fw: TCT CCT GGA AAT GTT GGC CCA TCT<br>Rv: AAT CCG ATG TTG CCA GCT TCA CCT
Cort (NM_001420759.1)	Fw: GCCTTCTGACTTTCCTTGCC<br>Rv: GAAAGCTCCCCGCTGATTGA
Drd1 (NM_001291801.1)	Rw: AGATGACTCCGAAGGCAGCCTT<br>Rv: GCCATGTAGGTTTTGCCTTGTGC
Gdnf (NM_001301332.1)	Fw: TCC AAC TGG GGG TCT ACG G<br>Rv: GCC ACG ACA TCC CAT AAC TTC AT
Gfap (NM_010277.3)	Fw: GGA GAT GCG GGA TGG TGA G<br>Rv: ACC ACG TCC TTG TGC TCC TG
Gpr88 (NM_001411424.1)	Fw: TGCGAGGAAGAAGAATCGTGGG<br>Rv: GCCATTCACGATGAAGGCGTTG
Mmp3 (NM_010809.3)	Fw: GACGATGATGAACGATGGACAG<br>Rv: CCTTGGCTGAGTGGTAGAGTC
Ghsr (NM_177330.4)	Fw: TCAGGGACCAGAACCACAAA<br>Rv: CCAGCAGAGGATGAAAGCAA
Iba1(NM_001409899.1)	Fw: GCT GGA GGG GAT CAA CAA GC<br>Rv: TCT TCA GCT CTA GGT GGG TCT
Il6 (NM_001314054.1)	Fw: TTC CAT CCA GTT GCC TTC TT<br>Rv: CAT TTC CAC GAT TTC CCA GA
Il10 (NM_010548.2)	Fw: GGT TGC CAA GCC TTA TCG GA<br>Rv: ACC TGC TCC ACT GCC TTG CT

Ocln (NM_001360538.1)	Fw: ATC CTG TCT ATG CTC ATT ATT GT<br>Rv: GCT GCT CTT GGG TCT GTA TAT CC
Rplp0 (NM_007475.5)	Fw: TGC ACT CTC GCT TTC TGG AG<br>Rv: CTG ACT TGG TTG CTT TGG CG
Scube3 (NM_001368734.1)	Fw: GCTGTGTCAACATGATGGGC<br>Rv: CCGCTGGATACAGGTATGCTG
Sstr1 (NM_001411890.1)	Fw:TGCCCTTTCTGGTCACTTCC<br>Rv:AGCGGTCCACACTAAGCACA
Sstr2 (NM_009217.5)	Fw:TGATCCTCACCTATGCCAACA<br>Rv:CTGCCTTGACCAAGCAAAGA
Sstr3 (NM_001411768.1)	Fw:GCCTTCTTCGGCCTCTACTT<br>Rv:GAATGCGACGTGATGGTCTT
Sstr4 (NM_009219.3)	Fw:AGGCTCGTGCTAATGGTGGT<br>Rv:GGATGAGGGACACATGGTTG
Sstr5 (NM_011425.3)	Fw:ACCCCCTGCTCTATGGCTTT<br>Rv:GCTCTATGGCATCTGCATCCT
Tnfα (NM_001278601.1)	Fw: GCG ACG TGG AAC TGG CAG AAG AG<br>Rv: TGA GAG GGA GGC CAT TTG GGA AC
Trem2 (NM_031254.4)	Fw: GCA CCT CCA GGA ATC AAG AG<br>Rv: GGG TCC AGT GAG GAT CTG AA

Table S1. Sequence of primers used for real-time PCR quantifications. Rv: reverse; Fw: forward. *Adora2a: adenosine A2a receptor; Arg1: arginase 1; Bdnf:* brain-derived neurotrophic factor*; Cst:* cortistatin*; Cd68:* CD68 antigen*; Cd163:* CD163 antigen*; Cd5l:* CD5 antigen-like*; Cldn1:* claudin 1*; Cldn5:* claudin 5*; Col1a2:* collagen type I alpha 2 chain*; Drd1:* dopamine receptor D1*; Gdnf:* glial cell line-derived neurotrophic factor*; Gfap: glial fibrillary acidic protein; Gpr88:* G protein-coupled receptor 88*; Mmp3:* matrix metallopeptidase 3*; Ghsr:* growth hormone secretagogue receptor*; Iba1:* allograft inflammatory factor 1*; Il6*: interleukin 6*; Il10:* interleukin 10*; Ocln:* occludin*; Rplp0:* ribosomal protein lateral stalk subunit P0*; Scube3:* signal peptide, CUB domain and EGF-like domain-containing protein 3*; Sst:* somatostatin*; Sstr1:* somatostatin receptor 1*; Sstr2:* somatostatin receptor 2*; Sstr3:* somatostatin receptor 3*; Sstr4:* somatostatin receptor 4*; Sstr5:* somatostatin receptor 5*; Tnfα:* tumor necrosis factor*; Trem2:* triggering receptor expressed on myeloid cells 2.

	Gene group	Description	Role in stroke (if upregulated)
**Immune response**	*Ccl12, Ccl9*	Recruit inflammatory cells to the site of injury	Worsen post-stroke inflammation
	Cd22, Cd52, Cd74	Immune cell regulation	Inflammatory response
	Irf7, Ifi44, Ifit1, Ifit3, Ifi30, Ifi213, Ifi207, Slfn9	Interferon-related genes; innate immune response; upregulated to combat cellular stress during ischemia	Prolonged inflammatory response post-stroke
	Gbp3, Oas1a, Oas1b, Oas2, Oasl2	Interferon-mediated immune responses	Neuroinflammation
	Spp1	Pro-inflammatory and pro-repair	Exacerbates immune activation
	Klk6	Immune modulation and extracellular matrix degradation	Can worsen neuroinflammation
	Evi2b	Immune cell activation	Heightened inflammatory signalling
	Siglec1	Macrophage activation	Increased inflammatory activity
	Fcgr1, Fcgr4	Fc gamma receptors that regulate immune cell activation	Heightened inflammation
	Isg15	Immune regulation	Worsens neuroinflammation
	Ddx60	Antiviral immune response	Worsens inflammation
	Usp18	Protein degradation and immune regulation	Post-stroke inflammation
	Gdf15	Neuroprotective cytokine	Anti-apoptotic and anti-inflammatory responses
**BBB/vascular**	*Mmp3*	Matrix metalloproteinases	BBB disruption and tissue remodelling
	Tpm4	Regulates actin cytoskeleton	Vascular remodelling
	Plaur	Modulates fibrinolysis	Vascular remodelling; angiogenesis
	Vim	Intermediate filament protein cytoskeletal remodelling	Reactive astrocytosis and BBB disruption
**Cell cycle**	*Ncapg, Ccnb2, Mis18bp1*	Cell cycle regulation	Increased cellular proliferation
	Emp1	Regulates cell proliferation and migration	Neurogenesis and vascular remodelling
**Neuronal**	*Nes*	Marker of neural stem cells	Neurogenesis
	Clcf1	Neurotrophic and anti-inflammatory effects	Neuroprotection
**Oxidative stress**	*Akr1b8*	Aldose reductase pathway; glucose metabolism	Response to oxidative stress; metabolic shift after stroke
	Dhrs1	Regulates lipid metabolism and oxidative stress	May be protective

	Msr1	Regulates lipid metabolism and inflammation	Chronic inflammation
**Unknown stroke evidence**	*Rnf213*	Associated with a cerebrovascular disorder that can lead to ischemic stroke	Unknown
	Apobec1	RNA editing in microglia	May be neuroprotective
	Atf3	Stress-inducible transcription factor	May be neuroprotective
	Fblim1	Cytoskeletal and focal adhesion regulation	May exacerbate post-ischemic inflammation
	Fosl1	Component of the AP-1 transcription factor complex	May be associated with pro-inflammatory signalling and glial activation
	Gbp5	Interferon-inducible GTPase involved in innate immunity	Strong type I interferon or inflammasome activity may contribute to neuroinflammation
	Ligp1	Cytosolic DNA sensor involved in innate immune responses	Activation of DNA damage or DAMP sensing pathways. Could promote inflammatory gene expression
	Kif14	Mitotic spindle motor protein involved in cell division	May reflect astrocyte or microglia proliferation
	Nlrc5	Transcriptional activator of MHC class I genes	May enhance immune surveillance or glial priming
	Phf11b	Transcriptional regulator involved in immune gene expression	May induce lymphocyte activation or interferon responses
	Trim30a	Negative regulator of NF-κB signalling	May act to dampen inflammation
	Uba7	Involved in ISGylation	May activate brain interferon-stimulated pathways

Table S11. Differentially expressed genes (DEGs) identified between healthy (CL) and ischemic (MCAO) hemispheres common in both genotypes with stronger upregulation in *Cort*$^{-/-}$ (depicted in Fig. 2d). “More up” genes in *Cort*$^{-/-}$ were described in terms of biological relevance and role in stroke when upregulated, and were classified into 6 main categories (*i.e.,* immune response, BBB and vascular dynamics, cell cycle and cell proliferation, brain and neuronal processes, oxidative stress, and unknown evidence for ischemic stroke).

	Gene group	Description	Role in stroke (if upregulated)
**Immune response**	*Ccl3, Ccl4, Ccl7*	Recruiting inflammatory cells to the site of injury	Worsen post-stroke inflammation; immune cell recruitment for debris clearance
	Ch25h	Regulates lipid metabolism and inflammation	Neuroinflammatory regulation
	Osm	Pleiotropic cytokine	Tissue remodelling, repair, angiogenesis
	Cd5l	Modulates macrophage function and anti-inflammatory responses	Tissue repair
	Pdcd1	Immune checkpoint inhibitor that regulates T-cell activity	May reduce excessive immune activation and limit neuroinflammation
	Cst7	Inhibitor of cysteine proteases	Limits excessive proteolytic activity, reducing secondary damage
	Phf11a	Transcription factor involved in immune responses	Post-stroke inflammation
**BBB/vascular**	*Mmp12*	Matrix metalloproteinase	Extracellular matrix degradation and inflammatory responses; favours tissue repair and vascular remodelling
	Htr2b	Serotonin receptor	Neuroprotection and improvement of cerebral blood flow
	Tgm1	Protein crosslinking	Tissue remodelling
**Cell cycle**	*Pimreg*	Regulates cell proliferation and survival	Neural and vascular cell regeneration; glial scarring
	Cep55	Regulates cell division and cytokinesis	Neural cell proliferation during recovery
	Cdca5	Controls cell cycle progression and DNA repair	Post-stroke brain tissue repair; enhances neurogenesis and cell survival
	Prr11	Regulates cell cycle and proliferation	Neurogenesis
**Neuronal**	*Troap*	Involved in cell adhesion and mitosis	Supports cell-cell interactions needed for brain repair and neural regeneration
**Unknown stroke role**	*Cd300ld*	STAT3-S100A8/A9 axis	Potentially involved in immune sensing and cleanup of cellular debris, with potential roles in resolution of inflammation
	Slamf7	Immune cell surface receptor involved in activating NK cells, macrophages, and some T and B cell subsets	May contribute to tissue damage if upregulated early, or to clearance of infected or damaged cells in later stages

Table S12. Differentially expressed genes (DEGs) identified between healthy (CL) and ischemic (MCAO) hemispheres common in both genotypes with stronger upregulation in *Cort*$^{+/+}$ (depicted in Fig. 2d). "More up" genes in *Cort*$^{+/+}$ were described in terms of biological relevance and role in stroke when upregulated, and were classified into 5 main categories (*i.e.,* immune response, BBB and vascular dynamics, cell cycle and cell proliferation, brain and neuronal processes, and unknown evidence for ischemic stroke).

	Gene group	Description	Role in stroke (if upregulated)	More down in
**Immune response**	*Cd4*	Marker and co-receptor for helper T cells	Reduced pro-inflammation; improved stroke outcomes; may impair clearance and repair	*Cort$^{-/-}$*
**BBB/vascular**	*Drd1*	Mediates dopaminergic signalling	Neurovascular regulation; limits neuroprotection; exacerbates endothelial hyperpermeability and BBB disruption; exacerbates neuroinflammation	*Cort$^{-/-}$*
**Unknown stroke evidence**	*Lrrc10b*	Regulation of the L-type calcium channel and excitation-contraction coupling	Neuroinflammatory responses; impair cellular resilience to ischemic stress; impair calcium homeostasis in cerebrovascular cells; BBB integrity	*Cort$^{-/-}$*
	Gpr88	Involved in striatal function and motor control, modulating dopaminergic and glutamatergic signalling	Dysregulation of motor control pathways and cognitive deficits post-stroke, as well as altered inflammatory responses	*Cort$^{-/-}$*
	Gng7	G-protein signalling	Disrupt neurovascular coupling, worsening ischemic damage and impairing recovery by altering GPCR-mediated protective pathways; exacerbates excitotoxicity	*Cort$^{-/-}$*
	Rgs9	Modulates GPCR signalling	Prolongs harmful excitatory signalling, increasing oxidative stress, inflammation, vascular dysfunction and neuronal injury	*Cort$^{-/-}$*
	Rasd2	GTP-binding protein	Impaired dopaminergic signalling and plasticity, limiting repair processes	*Cort$^{-/-}$*
	Ppp1r1b	Integrates dopaminergic and glutamatergic signalling in neurons	Disturbed signal integration, decreased neuroprotection, impaired learning/memory circuits	*Cort$^{-/-}$*
	Ppp3ca	Calcium/calmodulin-dependent phosphatase; activates NFAT pathway	Reduced synaptic remodelling, impaired neuroinflammation control	*Similar Cort$^{+/+}$ and Cort$^{-/-}$*
	Ppp3r1	Supports Ppp3ca in phosphatase activity	Suppresses calcineurin pathway, limiting recovery and repair	*Similar Cort$^{+/+}$ and Cort$^{-/-}$*
	Fbxl16	Part of SCF E3 ubiquitin ligase complex	Disruption in degradation of damaged proteins, leading to cellular stress	*Similar Cort$^{+/+}$ and Cort$^{-/-}$*
	Syndig1l	Synapse formation and neuronal connectivity	Synaptic dysfunction and hinder plasticity, worsening cognitive and motor impairments post-stroke	*Cort$^{-/-}$*
	Scube3	Angiogenesis and tissue repair	Impairs post-stroke vascular remodelling and neurovascular unit integrity, reducing recovery potential	*Cort$^{-/-}$*

	Ptprv	Negative regulator of cytokine signalling	Dysregulated immune signalling, potentially exacerbating inflammation or impairing resolution	*Cort$^{-/-}$*
	Scn4b	Modulates voltage-gated sodium channels	Potentially limiting excitotoxicity but impairing signalling	*Cort$^{-/-}$*
	Lzts3	Possible role in transcription regulation	Potential deregulation of cell cycle or stress response pathways	*Similar Cort$^{+/+}$ and Cort$^{-/-}$*
	Hpca	Neuronal calcium sensor protein	Altered calcium signalling, impacting neuroprotection and neurotransmission	*Cort$^{-/-}$*
	Hlf	bZIP transcription factor; modulates circadian rhythm and stress responses in the brain	Disrupted circadian regulation and neuroprotective gene transcription	*Cort$^{+/+}$*
	Nrgn	Calmodulin-binding protein	Impaired synaptic plasticity, reduced neuronal adaptability post-injury	*Cort$^{+/+}$*
	Kcnab1	Regulates potassium channel kinetics	Excitotoxicity	*Similar Cort$^{+/+}$ and Cort$^{-/-}$*
	Foxp1	Transcription factor involved in neurodevelopment, synaptic transmission, and inflammation regulation	Impaired gene expression for neuroprotection and plasticity	*Similar Cort$^{+/+}$ and Cort$^{-/-}$*

Table S13. Downregulated differentially expressed genes (DEGs) identified between healthy (CL) and ischemic (MCAO) hemispheres, common in both genotypes (depicted in Fig. 2d). “More down” genes are showed for each genotype. Genes were described in terms of biological relevance, role in stroke when downregulated, and were classified into 3 main categories (*i.e.,* immune response, BBB and vascular dynamics, and unknown evidence for ischemic stroke).

Age-Time post-stroke	Genotype	Lesion size ≥ 30 mm^3	Lesion size < 30 mm^3
Young-48h (acute)	*Cort*$^{+/+}$ (N = 9)	0	7 (100%)
	Cort$^{-/-}$ (N = 8)	3 (37.5%)	5 (62.5%)
	Cort$^{-/-}$ + CST (N = 7)	2 (28.6%)	5 (71.4%)
Young-7 days (subacute)	*Cort*$^{+/+}$ (N=5)	0	5 (100%)
	Cort$^{+/+}$ + CST (N=5)	0	5 (100%)
	Cort$^{-/-}$ (N=5)	1 (20%)	4 (80%)
	Cort$^{-/-}$ + CST (N=6)	0	6 (100%)
Middle-aged-48h (acute)	*Cort*$^{+/+}$ (N=6)	0	6 (100%)
	Cort$^{+/+}$ + CST (N=6)	0	6 (100%)
	Cort$^{-/-}$ (N=5)	0	5 (100%)
	Cort$^{-/-}$ + CST (N=6)	0	6 (100%)
	**Genotype**	**$MAP2^-$ area ≥ 5 mm^2**	**$MAP2^-$ area < 5 mm^2**
Young-48h (acute)	*Cort*$^{+/+}$ (N = 8)	1 (12.5%)	7 (87.5%)
	Cort$^{-/-}$ (N =8)	6 (75%)	2 (25%)
	Cort$^{-/-}$ + CST (N = 5)	0	5 (100%)
Young-7 days (subacute)	*Cort*$^{+/+}$ (N=4)	0	4 (100%)
	Cort$^{+/+}$ + CST (N=4)	1 (25%)	3 (75%)
	Cort$^{-/-}$ (N=6)	1 (16.7%)	5 (83.3%)
	Cort$^{-/-}$ + CST (N=6)	1 (16.7%)	5 (83.3%)
Middle-aged-48h (acute)	*Cort*$^{+/+}$ (N=5)	3 (60%)	2 (40%)
	Cort$^{+/+}$ + CST (N=5)	0	5 (100%)
	Cort$^{-/-}$ (N=5)	2 (40%)	3 (60%)
	Cort$^{-/-}$ + CST (N=6)	0	6 (100%)

Table S15. Quantification of neurological lesion. For each group, data indicate the absolute number and percentage of total animals with large lesion (over 30 mm^3) and significant neuronal loss (MAP2 negative area over 5 mm^2). *Cort*$^{+/+}$: *Cort*$^{+/+}$ MCAO saline-treated mice, *Cort*$^{-/-}$ + CST: *Cort*$^{+/+}$ MCAO cortistatin-treated mice, *Cort*$^{-/-}$: *Cort*$^{-/-}$ MCAO saline-treated mice, and *Cort*$^{-/-}$ + CST: *Cort*$^{-/-}$ MCAO cortistatin-treated mice.

Age-Time post-stroke	Genotype	0 reaches (WH)	WH Reaches > 0
Young-48h (acute)	*Cort*$^{+/+}$ (N = 10)	2 (20%)	8 (80%)
	Cort$^{-/-}$ (N = 9)	4 (44.4%)	5 (55.6%)
	Cort$^{-/-}$ + CST (N = 7)	2 (28.6%)	5 (71.4%)
Young-5 days (subacute)	*Cort*$^{+/+}$ (N=5)	0	5 (100%)
	Cort$^{+/+}$ + CST (N=5)	0	5 (100%)
	Cort$^{-/-}$ (N=5)	2 (40%)	3 (60%)
	Cort$^{-/-}$ + CST (N=7)	3 (42.8%)	4 (57.2%)
Middle-age-48h (acute)	*Cort*$^{+/+}$ (N=5)	2 (40%)	3 (60%)
	Cort$^{+/+}$ + CST (N=8)	5 (62.5%)	3 (37,5%)
	Cort$^{-/-}$ (N=4)	3 (75%)	1 (25%)
	Cort$^{-/-}$ + CST (N=7)	5 (71.4%)	2 (28.6%)
	**Genotype**	**WH falls ≥ 5**	**WH falls < 5**
Young-48h (acute)	*Cort*$^{+/+}$ (N = 10)	0	10 (100%)
	Cort$^{-/-}$ (N =10)	4 (40%)	6 (60%)
	Cort$^{-/-}$ + CST (N = 9)	0	9 (100%)
Young-5 days (subacute)	*Cort*$^{+/+}$ (N=6)	0 (0%)	6 (100%)
	Cort$^{+/+}$ + CST (N=6)	0 (0%)	6 (100%)
	Cort$^{-/-}$ (N=6)	0 (0%)	6 (100%)
	Cort$^{-/-}$ + CST (N=7)	4 (42.8%)	3 (57.2%)
Middle-aged-48h (acute)	*Cort*$^{+/+}$ (N=6)	2 (33.3%)	4 (66.67%)
	Cort$^{+/+}$ + CST (N=10)	7 (70%)	3 (30%)
	Cort$^{-/-}$ (N=5)	4 (80%)	1 (20%)
	Cort$^{-/-}$ + CST (N=9)	9 (100%)	0
	**Genotype**	**Time to descend > 20 s**	**Time to descend < 20 s**
Young-48h (acute)	*Cort*$^{+/+}$ (N = 9)	3 (33,3%)	6 (66.7%)
	Cort$^{-/-}$ (N = 6)	2 (33.3%)	4 (66.7%)
	Cort$^{-/-}$ + CST (N = 5)	1 (20%)	4 (80%)
Young-5 days (subacute)	*Cort*$^{+/+}$ (N=4)	2 (50%)	2 (50%)
	Cort$^{+/+}$ + CST (N=6)	3 (50%)	3 (50%)
	Cort$^{-/-}$ (N=7)	4 (57.2%)	3 (42.8%)
	Cort$^{-/-}$ + CST (N=6)	3 (50%)	3 (50%)
Middle-aged-48h (acute)	*Cort*$^{+/+}$ (N=4)	1 (25%)	3 (75%)
	Cort$^{+/+}$ + CST (N=7)	4 (57.2%)	3 (42.8%)
	Cort$^{-/-}$ (N=4)	1 (25%)	3 (75%)
	Cort$^{-/-}$ + CST (N=6)	0	6 (100%)

Table S16. Quantification of neurofunctional deficits. For each group, data indicate the absolute number and percentage of total animals with no reaches and more than 5 falls from the wire-hanging test (WH), as well as animals categorized based on the time taken to descend from the pole test. *Cort*$^{+/+}$: *Cort*$^{+/+}$ MCAO saline-treated mice, *Cort*$^{-/-}$ + CST: *Cort*$^{+/+}$ MCAO cortistatin-treated mice, *Cort*$^{-/-}$: *Cort*$^{-/-}$ MCAO saline-treated mice, and *Cort*$^{-/-}$ + CST: *Cort*$^{-/-}$ MCAO cortistatin-treated mice.
